# Molecular surveillance of anti-malarial resistance *pfcrt, pfmdr1,* and *pfk13* polymorphisms in African *Plasmodium falciparum* imported parasites to Wuhan, China

**DOI:** 10.1186/s12936-021-03737-8

**Published:** 2021-05-01

**Authors:** Weijia Cheng, Xiaonan Song, Huabing Tan, Kai Wu, Jian Li

**Affiliations:** 1 Department of Human Parasitology, School of Basic Medical Sciences, Hubei University of Medicine, Shiyan, 442000 China; 2 Department of Infectious Diseases, Renmin Hospital, Hubei University of Medicine, Shiyan, 442000 China; 3 Department of Schistosomiasis and Endemic Diseases, Wuhan City Center for Disease Prevention and Control, Wuhan, 430024 China

**Keywords:** Imported malaria, *Plasmodium falciparum*, Anti-malarial resistance, Molecular surveillance, Wuhan

## Abstract

**Background:**

Imported malaria parasites with anti-malarial drug resistance (ADR) from Africa is a serious public health challenge in non-malarial regions, including Wuhan, China. It is crucial to assess the ADR status in African *Plasmodium falciparum* isolates from imported malaria cases, as this will provide valuable information for rational medication and malaria control.

**Methods:**

During 2017–2019, a cross-sectional study was carried out in Wuhan, China. Peripheral blood 3 ml of returned migrant workers from Africa was collected. The target fragments from *pfcrt*, *pfmdr1*, and *k13 propeller* (*pfk13*) genes were amplified, sequenced, and analysed.

**Results:**

In total, 106 samples were collected. Subsequently, 98.11% (104/106), 100% (106/106), and 86.79% (92/106) of these samples were successfully amplified and sequenced for the *pfcrt* (72–76), *pfmdr1*, and *pfk13* genes, respectively. The prevalence of the *pfcrt* 76** T**, *pfmdr1* 86**Y**, and *pfmdr1* 184**F** mutations was 9.62, 4.72, and 47.17%, respectively. At codons 72–76, the *pfcrt* locus displayed three haplotypes, CVMNK (wild-type), CV**IET** (mutation type), CV M/**I** N/**E** K/**T** (mixed type), with 87.50%, 9.62%, and 2.88% prevalence, respectively. For the *pfmdr1* gene, NY (wild type), N**F** and **YF** (mutant type), N Y/**F**, Y Y/**F**, and N/**Y** Y/**F** (mixed type) accounted for 34.91, 43.40, 3.77, 15.09, 0.94, and 1.89% of the haplotypes, respectively. A total of 83 isolates with six unique haplotypes were found in *pfcrt* and *pfmdr1* combined haplotypes, of which NY-CVMNK and N**F**-CVMNK accounted for 40.96% (34/83) and 43.37% (36/83), respectively. Furthermore, 90 cases were successfully sequenced (84.91%, 90/106) at loci 93, 97, 101, and 145, and 78 cases were successfully sequenced (73.58%, 78/106) at loci 343, 353, and 356 for *pfcrt*. However, the mutation was observed only in locus 356 with 6.41%. For *pfk13*, mutations reported in Southeast Asia (at loci 474, 476, 493, 508, 527, 533, 537, 539, 543, 553, 568, 574, 578, and 580) and Africa (at loci 550, 561, 575, 579, and 589) were not observed.

**Conclusions:**

The present data from *pfcrt* and *pfmdr1* demonstrate that anti-malarial drugs including chloroquine, amodiaquine, and mefloquine, remain effective against malaria treatment in Africa. The new mutations in *pfcrt* related to piperaquine resistance remain at relatively low levels. Another source of concern is the artemether-lumefantrine resistance-related profiles of N86 and 184**F** of *pfmdr1*. Although no mutation in *pfk13* is detected, molecular surveillance must continue.

**Supplementary Information:**

The online version contains supplementary material available at 10.1186/s12936-021-03737-8.

## Background

Malaria is a mosquito-borne infectious disease that seriously threatens human health, among which falciparum malaria caused by *Plasmodium falciparum* is the most serious, mainly in tropical and subtropical regions in sub-Saharan Africa and Southeast Asia (SEA) [1]. In 2019, there was an estimated 229 million malaria cases from 87 malaria-endemic countries. Furthermore, approximately 94% of estimated cases were detected in Africa. The countries of Nigeria, Congo, Uganda, Mozambique, and Niger account for 51% of malaria cases (117 million). Additionally, it estimated approximately 409,000 deaths are estimated globally [1]. Although there have been no indigenous malaria cases reported in China for three consecutive years since 2017 [2], potential challenges remain in imported malaria cases. In recent years, with globalization, the number of migrant workers, tourists, and businesspeople in China has increased gradually, especially those returning from Africa and SEA [3], which has brought severe pressure for malaria eradication in China. Thus, it is necessary to strengthen surveillance for imported malaria.

Anti-malarial drugs are considered the major measure for malaria control [4]. However, with the continuous use of anti-malarial drugs, *P. falciparum* gradually achieves drug resistance and spreads rapidly [5]. Chloroquine (CQ) is a safe, inexpensive, and effective anti-malarial drug for malaria therapy. However, in the 1940s, *P. falciparum* parasites developed resistance to CQ. Since then, CQ-resistant (CQR) strains have begun to spread rapidly around the world [6]. After the discovery of artemisinin (ART) in the 1970s, malaria control was temporarily eased. To improve clinical efficacy and delay the emergence of parasite drug resistance, artemisinin-based combination therapy (ACT) have been recommended by the World Health Organization (WHO) since 2001 [7]. Unfortunately, ART resistance of *P. falciparum* isolates was reported in SEA [8–10]. Recently, dihydroartemisinin-piperaquine (DHA-PPQ) resistance has been detected in western Cambodia [11–14]. Although ACT remains effective in Africa and SEA, prolonged use of ART would lead to anti-malarial drug resistance. Anti-malarial drug resistance (ADR) would be disastrous for global malaria control. Therefore, in the absence of more choices, it is urgent to monitor the ADR status of *P. falciparum* parasites.

Mutations detected in *P. falciparum* essential genes including *pfcrt*, *pfmdr1*, *pfdhfr*, *pfdhps*, *pfk13,* and *pfpm2* have been used as molecular markers of drug resistance. The *pfk13* polymorphism has been considered to be related to ART resistance [9]. However, previous studies demonstrated that the distribution of alleles for *pfk13* varies according to the mutations [15]. In SEA, the alleles of the 580**Y** mutation account for the vast majority [9]. In Africa, the mutation rate of *pfk13* remained relatively low. In 2016, the newly discovered local ART resistance mutation 561**H** of *pfk13* was reported from Rwanda, Africa [16]. The 72–76 amino acid mutation in *pfcrt*, especially the 76** T** mutation, was the primary marker of CQR [17–19]. Several mutations in *pfmdr1* are related to the resistance of *P. falciparum* to CQ, amodiaquine (AQ) and mefloquine (MQ) [20, 21]. At present, several newly detected mutations in *pfcrt,* including 93**S**, 97**Y**, 101**F**, 145**I**, 343**L**, 353** V** and 356** T**, have been identified to be associated to with PPQ with a decreasing trend for the susceptibility of *P. falciparum* strains in South America [13]. However, there was limited information on the effects of these alleles on PPQ in Africa, where malaria is endemic.

In the present study, polymorphisms of *pfcrt*, *pfmdr1*, and *pfk13* for *P. falciparum* isolates imported from Africa in Wuhan, China were surveyed. This survey will provide valuable information for rational medication for malaria patients in clinical practice, preventing the spread of ADR *P. falciparum* in Africa and China.

## Methods

### Collection of samples

A cross-sectional study was performed in Wuhan, Hubei Province, China. Peripheral blood 3 ml of returned migrant workers from African countries was collected at major hospitals in the region from May 2017 to December 2019. These samples were collected from Wuhan Jinyintan Hospital, Wuhan Union Hospital and Zhongnan Hospital of Wuhan University, and Tongji Hospital. These samples were examined by an immuno-gold assay kit (ICT Diagnostics) for *Plasmodium spp.* antigen (HRPII). Then, blood smears were prepared and checked under a microscope. Finally, the species were identified by qPCR. Approximately 400 μl of blood was spotted on 3 MM Whatman filter paper and air-dried (identified and provided by the Center for Disease Control and Prevention of Wuhan City, Hubei Province). Then, these filter papers were numbered and stored at − 20 °C with in polyethylene bag. Consent of the owner and his legal guardian was obtained before sampling.

### Determination of *Plasmodium falciparum* gene mutations

Genomic DNA (gDNA) was extracted from blood-spots by a TIANamp Blood Spots DNA Kit to yield approximately 50 μl of supernatant containing gDNA and stored at − 20 °C until further use. The target fragments of the *pfcrt*, *pfmdr1*, and *pfk13* genes were amplified from the gDNA sample via nested PCR and traditional PCR. Following previously published primer information and a previous procedure [13, 16, 22, 23], the *pfcrt*, *pfmdr1*, and *pfk13* genes were successfully amplified. The reaction system and procedure for PCR are listed in Additional file 2: Table S1. Loci 72–76 of the *pfcrt* gene and *pfmdr1* gene were subjected to two rounds of PCR amplification (nested PCR). Loci 93–356 of the *pfcrt* gene were subjected to one round of PCR amplification (traditional PCR). After all the reactions finished, the 5.0 μl PCR products were analysed by 1.0% agarose gel electrophoresis. Then the remaining products were purified for Sanger sequencing (Genewiz, Soochow, China). The reference sequences of the *P. falciparum* 3D7 strain were downloaded from the database PlasmoDB (http://plasmodb.org/plasmo/) with the gene IDs: PF3D7_0709000 (*pfcrt*), PF3D7_0523000 (*pfmdr1*), and PF3D7_1343700 (*pfk13*). The sequencing data were analysed with Dnastar (DNASTAR Inc., Madison, WI, USA) and compared with the standard sequence. Synonymous mutations and nonsynonymous mutations were detected, and confirmed via traditional bidirectional Sanger sequencing. To avoid any kind of technical contamination, plasmids with known mutant alleles of *pfcrt*, *pfmdr1*, and *pfk13* were used as the positive controls for these samples (Additional file 1: Fig. S1).

### Data analysis

Excel software (Microsoft Excel; version 2016) was used to record the data and calculate the frequency of single nucleotide polymorphisms (SNPs) and haplotypes. The 95% confidence intervals of wild-type and mutant types for these genes were analysed with SPSS 25 (SPSS Inc., Chicago, IL, USA). Mixed-type genes, which include mixed infections of wild-type and mutant type, were excluded from the combined haplotype analysis [16].

## Results

### General information

A total of 106 of these returnees were diagnosed with *P. falciparum* from 2017 to 2019, all from Africa (Additional file 3: Table S2), including 48 cases in West Africa, 33 cases in Central Africa, 15 cases in South Africa, nine cases in East Africa, and one case in North Africa. These cases were mainly from 21 African countries, particularly concentrated in Congo (21.70%, 23/106), followed by Nigeria (16.98%, 18/106), Ivory Coast (9.43%, 10/106), and Mozambique (6.60%, 7/106).

### Mutation prevalence of *pfcrt* and *pfmdr1*

The polymorphisms and haplotypes of *pfcrt* and *pfmdr1* were analysed. For the *pfcrt* mutations C72**S**, M74**I**, N75**E**, and K76**T**, a total of 104 samples were successfully sequenced (98.11%, 104/106). Codon 72 in all samples had 100% wild type, and the mutation frequency of codons 74, 75, and 76 was 9.62% (10/104) (Table 1). These mutations were mainly concentrated in West Africa, followed by Central Africa. The results showed that the *pfcrt* genotype had polymorphisms at codons 72–76, including CVMNK (wild-type), CV**IET** (mutation type), and CV M/**I** N/**E** K/**T** (mixed type). Most isolates harboured parasites with the CVMNK (87.5%, 91/104). The proportion of CV**IET** among these isolates was 9.62% (10/104). The last case was CV M/**I** N/**E** K/**T**, accounting for 2.88% (3/104), and no haplotype of **S**VMN**T** was found (Additional file 4: Table S3).

**Table 1:** Observed overall frequency of mutations in pfcrt and pfmdr1

Gene	Mutations	Wild type(%, 95% CI)	Mutation(%, 95% CI)	Mixed type(%, 95% CI)	Total
*pfcrt*	C72**S**	104(100.00, 1.00–1.00)	0(0.00, 0.00–0.00)	0(0.00, 0.00–0.00)	104
	M74**I**	91(87.5, 0.81–0.94)	10(9.62, 0.039–0.15)	3(2.88, − 0.004 to 0.062)	104
	N75**E**	91(87.5, 0.81–0.94)	10(9.62, 0.039–0.15)	3(2.88, − 0.004 to 0.062)	104
	K76**T**	91(87.5, 0.81–0.94)	10(9.62, 0.039–0.15)	3(2.88, − 0.004 to 0.062)	104
	I356**T**	70(89.74, 0.39–0.55)	5(6.41, 0.09–0.120)	3(3.84, − 0.005 to 0.082)	78
*pfmdr1*	N86**Y**	99(93.40, 0.89–0.98)	5(4.72, 0.006–0.088)	2(1.89, − 0.007 to 0.045)	106
	Y184**F**	37(34.91, 0.26–0.44)	50(47.17, 0.38–0.57)	19(17.92, 0.11–0.25)	106

Mutations are shown in underline and bold

**Fig. 1:**
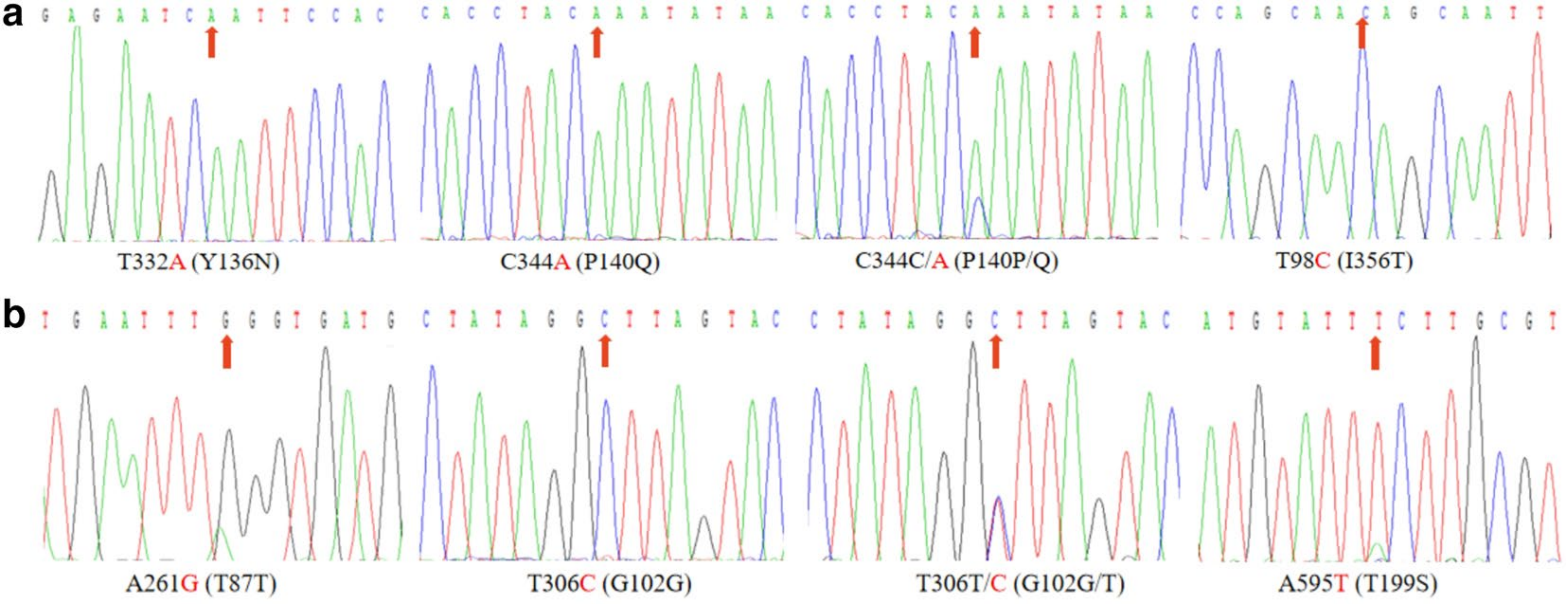
Sequence profile of PCR products with new mutation site in *pfmdr1* and *pfcrt* genes***.*** **a** The new mutation site detected in *pfmdr1* gene; **b** The mutation site found in *pfcrt* gene

For several new alleles of *pfcrt* (Additional file 3: Table S2), 90 cases were successfully sequenced (84.91%, 90/106) at loci 93, 97, 101 and 145, and no mutation was found. Additionally, two isolates (2.22%, 2/90) were found to have mutations at locus 136 (Fig. 1a); these isolates come from Nigeria (1.11%, 1/90) of West Africa and Mozambique (1.11%, 1/90) in South Africa. At loci 140 (Fig. 1a), one isolate carried mutant allele and 1 isolate (1.28%) was mixed, from Gabon (1.11%, 1/90), Central Africa, Nigeria (1.11%, 1/90), and West Africa. In addition, 78 cases were successfully sequenced (73.58%, 78/106) at loci 343, 350, 353, and 356. No mutations were observed at loci 343, 350, and 353. For polymorphisms at locus 356, 70 isolates (89.74%) carried the wild-type allele, five isolates (6.41%) carried the mutant allele, and three isolates (3.84%) were mixed.

For the *pfmdr1* gene, 100% of samples were obtained from the PCR products and sequenced successfully (Table 1). Sequencing data illustrated that the main epidemic mutation sites of *pfmdr1* were concentrated at 86**Y** and 184**F**, with 4.72% and 47.17% mutations, respectively (Table 1). These mutations were also mainly focused in West Africa, followed by Central Africa and South Africa. Additionally, six haplotypes were detected in *pfmdr1* gene (Additional file 4: Table S3), including NY (wild type), N**F** and Y**F** (mutant type), NY/**F**, **Y**Y/**F**, and N/**Y** Y/**F** (mixed type), accounting for 34.91, 43.40, 3.77, 15.09, 0.94, and 1.89%, respectively. In addition, one nonsynonymous mutation at position 199 and several synonymous mutations at positions 87 and 102 were detected in these samples (Table 2; Fig. 1b).

**Table 2:** Novel polymorphisms of pfmdr1 and pfcrt in* Plasmodium falciparum* isolates

Gene	Reference^*a*^			Mutation^*b*^			Number of isolates			
	Codon position	AA^*c*^	Codon	AA^*c*^	Codon	Base position	PCR positive	Sequencing	Mutation	Prevalence (%, 95% CI)
*pfmdr1*	87	L	tt**a**	L	tt**G**	261	106	106	1	0.94(− 0.009 to 0.028)
	102	G	gg**t**	G	gg**C**	306			2	1.89(− 0.007 to 0.045)
	102	G	gg**t**	G	gg**C/T**	306			1	0.94(− 0.009 to 0.028)
	199	T	**a**ct	S	**T**ct	595			1	0.94(− 0.009 to 0.028)
*pfcrt*	136	Y	**t**at	N	**A**at	332	90	90	2	2.22(− 0.009 to 0.053)
	140	Q	c**a**a	K	c**C**a	344			1	1.11(− 0.011 to 0.033)
	140	Q	c**a**a	Q/K	c**A/C**a	344			1	1.11(− 0.011 to 0.033)

^a^Reference site is in bold type with lowercase

^b^Mutation site is in bold type with capital letter

^c^*AA* amino acid residue

With these polymorphisms in the *pfcrt* and *pfmdr1* genes, a total of six *pfcrt*/*pfmdr1* combined haplotypes were assessed, namely, N**F**-C**VIET**, N**F**-CVMNK, NY-CV**IET**, NY-CVMNK, **YF**-CV**IET**, and **YF**-CVMNK, accounting for 7.23% (6/83), 43.37% (36/83), 3.61% (3/83), 40.96% (34/83), 1.20% (1/83), and 3.61% (3/83), respectively (Additional file 5: Table S4). These haplotypes were mainly concentrated in West Africa and Central Africa. Among them, Congo and Nigeria accounted for 21.69% (18/83) and 16.87% (14/83), respectively (Additional file 3: Table S2).

### Analysis of mutation in *pfk13* gene

For the *pfk13*, 93.40% (99/106) samples were amplified, and 92.93% (92/106) of the samples were sequenced successfully. Sequencing analysis showed that these isolates were all wild-type. The reported mutations in SEA at loci 474, 476, 493, 508, 527, 533, 537, 539, 543, 553, 568, 574, 578, and 580 were not detected in the current study. Furthermore, the previously detected mutations at positions 550, 561, 575, 579, and 589 in Africa were also not found.

## Discussion

For the past several decades, the emergence and rapid transmission of *P. falciparum* ADR parasites has become a major cause of malaria burden globally [24]. In China, the continuous influx of imported malaria increases the possibility of malaria respreading [25]. The malaria-endemic area, including Africa and SEA, was the primary source of imported malaria in China including in Wuhan [16]. Thus, continuous surveillance of imported malaria and ADR profiles is essential for malaria eradication in the non-malarial regions, particularly Wuhan, China.

The mutation of 76** T** in *pfcrt* was related to CQR [17]. For *pfcrt*, CV**IET** and **S**VMN**T** were the dominant mutant haplotypes. In Africa, mutant haplotype CV**IET** occurs more frequently [26]. CVIET (9.62%) was the most common mutant haplotype in the current study and was mainly distributed in West Africa (5.77%). **S**VMN**T** is mainly detected in South America and SEA and is rarely found in Africa [27]. The presence of **S**VMN**T** was not found in this survey. However, **S**VMN**T** was observed in Tanzania and Angola [28, 29]. In these regions, AQ was considered as the driving factor for haplotype selection of **S** VMN**T** [28, 29]. The CQ treatment resulted in high failure rates in southern Cameroon between 1999 and 2004 [30]. However, after an interval of 9 years, the frequency of CVMNK in southeastern Cameroon nearly doubled; Conversely, the CV**IET** decreased significantly [31]. Drug pressure caused by CQ declined during the period as a result of the cessation of drug imports to these countries. In the present study, haplotypes of CVMNK, CV**IET**, CV M/**I** N/**E** K/**T** with proportions of 87.50, 9.62, and 2.88% were observed during 2017–2019, respectively. Compared with the previous study [16], all current observed data indicate that the wild-type haplotype is increased and haplotypes of the mutation type and mixed type are decreased. In recent years, the frequency of CVMNK has increased in several regions of Africa [32, 33], which is consistent with this survey results. In the present data, CVMNK is mainly concentrated in West Africa (36.54%) and Central Africa (28.85%), especially in the Congo (21.15%) and Nigeria (15.38%). After CQ was discontinued in most countries in sub-Saharan Africa in the 1990s, the investigated isolates regained all or part of their sensitivity to anti-malarial drugs [34, 35]. It will offer the possibility for these areas to reintroduce CQ in the future for malaria control. Therefore, continuous monitoring of *pfcrt* to evaluate CQ resistance dynamics in a certain area is urgent.

DHA/PPQ is one of the ACT, effective against simple malaria. Thus, the effect of PPQ cannot be ignored. However, long-term use of anti-malarial drugs particularly PPQ induced ADR [14]. Previous studies indicated that several mutations of *pfcrt* (93**S**, 97**Y**, 101**F**, 145**I**, 343**L**, 353** V**, and 356** T**) were related to reducing parasite sensitivity to PPQ [13, 14, 36–39]. In this study, no mutations were detected at loci 93, 97, 101, 145, 343, 350, and 353. In an investigation of African isolates, consistent with the results of this study, no mutations at these sites were reported [13]. In this study, 5 isolates carried the mutant allele, and 3 isolates were mixed type at loci 356. In 2011–2012, the 356** T** in Gambia and Congo were 78.7 and 36.5%, respectively [40]. The 356** T** mutation was found in 54.7% of *P. falciparum* detected in Africa in 2017–2018. However, they also reported that the 356** T** mutation was not associated with in vitro reduced susceptibility to PPQ [13]. Therefore, continuous observations of *pfcrt* mutations and susceptibility tests in vitro related to PPQ are necessary.

The *pfmdr1* gene has been reported to be involved in regulating drug sensitivity or tolerance to several anti-malarial drugs, such as CQ, MQ, quinine (QN), artemether-lumefantrine (AL), and even ART [41]. The *pfmdr1* gene 86**Y** mutation is a potential marker for CQR, while 184**F** may play a role in resistance to multiple anti-malarial drugs [41]. The previously reported 86**Y** and 184**F** mutations in *pfmdr1* are most prevalent in Asia and Africa [42]. The frequencies of 86**Y** (4.72%) and 184**F** (47.17%) were monitored in this study, of which 184**F** was more prevalent. The results were similar to previous results in Nigeria and Senegal [43, 44]. In addition, compared with this previous survey in 2011–2016 [16], allele 86**Y** was significantly reduced. This is consistent with the results discussed above regarding the sensitivity of *pfcrt* gene recovery to CQ in recent years. Among the six observed haplotypes in this study, N**F** (43.40%) and NY (34.91%) were also the most frequent, mainly found in West Africa and Central Africa, especially in Congo and Nigeria, which could be a result of selective pressure by resistance to different drugs. In Nigeria, a previous study showed that N**F** was closely related to the sensitivity of AL [45]. It may be that the first-line drug CQ is replaced by AL, leading to an increased incidence of N**F** in these countries.

The *pfk13* gene was crucial in the molecular surveillance of ADR for falciparum malaria parasites. To date, more than 200 nonsynonymous mutations of *pfk13* have been reported [46]. In SEA and, more recently, South America, a number of these mutations have been associated with delayed parasite clearance following ACT, including mutations at loci 446, 458, 474, 476, 493, 508, 527, 533, 537 543, 553, 568, 574, 580 and so forth [46]. In Africa, a number of nonsynonymous mutations in *pfk13* have been identified, including mutations at loci149, 189, 189, 561, 575, 579, 589, 578, 592, 637, 641, 656 and so forth [46, 47]. In this survey, no mutation was found in *pfk13*. Because these sample size was insufficient, it was not sufficient to say that the African plasmodium isolates were still highly sensitive to ART; it is necessary to carry out relevant tests with a larger sample size in the future. Although there is no mutation in the *pfk13* gene to indicate ART resistance, it cannot be ignored that *pfk13* is no longer the only biomarker of ART resistance, and there may be other genes as markers of ART resistance [48, 49]. Thus, genetic markers of ADR are urgently required. Previous studies have demonstrated that the new candidates *pfubp-1* and *pfap2mu* are implicated in ART resistance in the *P. falciparum* [48, 49]. Alarming the high morbidity and mortality rates in Africa and the increased status of ADR in Africa could hamper malaria prevention, control, elimination, and even eradication. Therefore, it is critical to monitor mutations associated with ART resistance globally, especially in Africa, by delaying parasite clearance.

It is worth noting that several shortcomings of the current study cannot be neglected. First, affected by the epidemic of COVID-19, the sample size remains small. Thus, valuable information for the molecular surveillance of ADR is limited. Second, 17 samples failed SNPs analysis of *pfk13* because of the failure of amplification and sequencing. In a further study, advanced gene-editing tools, particularly the CRISPR/Cas9 technique, should be considered using *P. falciparum* drug resistance genes [50, 51]. The CRISPR/Cas9 technique can rapidly locate the key sites related to ADR in targeted genes. Compared to natural mutation under long-term drug pressure, artificially introduced mutation by CRISPR/Cas9 can effectively shorten the process of discovering drug resistance sites. CRISPR/Cas9 will offer a useful measure for the discovery of novel mutations in drug resistance genes.

## Conclusions

Overall, this study analysed the frequency and spatial distribution of mutations associated with ADR in the *pfcrt*, *pfmdr1,* and *pfk13* genes from imported *P. falciparum* isolates in Wuhan, China. The wild-type *pfcrt* allele and *pfmdr1* N86 were predominant in this study. These phenomena indicate that the cessation of CQ, AQ and MQ for a period of time may lead to the restoration of CQ, AQ and MQ sensitive parasites (at least partially). Moreover, it these drugs can continue to be effective for *P. falciparum* malaria case treatment in Africa. However, the increase in N86 and 184**F** mutations suggests a potential risk of drug pressure in AL. For new alleles with reduced sensitivity of *pfcrt* to PPQ, the expansion of these mutations further demonstrates their more significant survival advantage under strong and sustained PPQ pressure. If the development continues, this will lead to the failure of the first-line regimen DHA-PPQ. Thus, constant and careful worldwide surveillance for PPQ resistance is urgent. Although no mutation is detected in *pfk13*, caution should be made regarding ART therapy for *P. falciparum* in Africa, and continuous molecular surveillance is still urgently necessary.

## Supplementary Information

**Additional file 1**: **Fig. S1**. Positive controls. A. pfcrt CVIET plasmid running in agarose gel electrophoresis and product sequence analysis; B. pfmdr1 NY plasmid with mutations at 86 and 184 sites running in agarose gel electrophoresis and product sequence analysis. C. Sequencing profile of pfk13 plasmid (JPG 3459 KB)

**Additional file 2**: **Table S1.** Reaction system and conditions for amplification of targeted fragments of pfmdr1 and pfcrt genes.

**Additional file 3**: **Table S2.** Haplotypes distribution of pfcrt and pfmdr1 during 2017-2019.

**Additional file 4**: **Table S3.** Haplotypes distribution of pfcrt and pfmdr1 from different areas.

**Additional file 5**: **Table S4.** Overall frequency of combined haplotypes in pfcrt and pfmdr1.

## Data Availability

The datasets analysed in this study are available from the corresponding author on reasonable request.
