# Supplementary figures and images for: Molecular surveillance of anti-malarial resistance pfcrt, pfmdr1, and pfk13 polymorphisms in African Plasmodium falciparum imported parasites to Wuhan, China
Source: Malar J. 2021 May 1;20:209. doi: 10.1186/s12936-021-03737-8 (PMC8087876; doi:10.1186/s12936-021-03737-8)

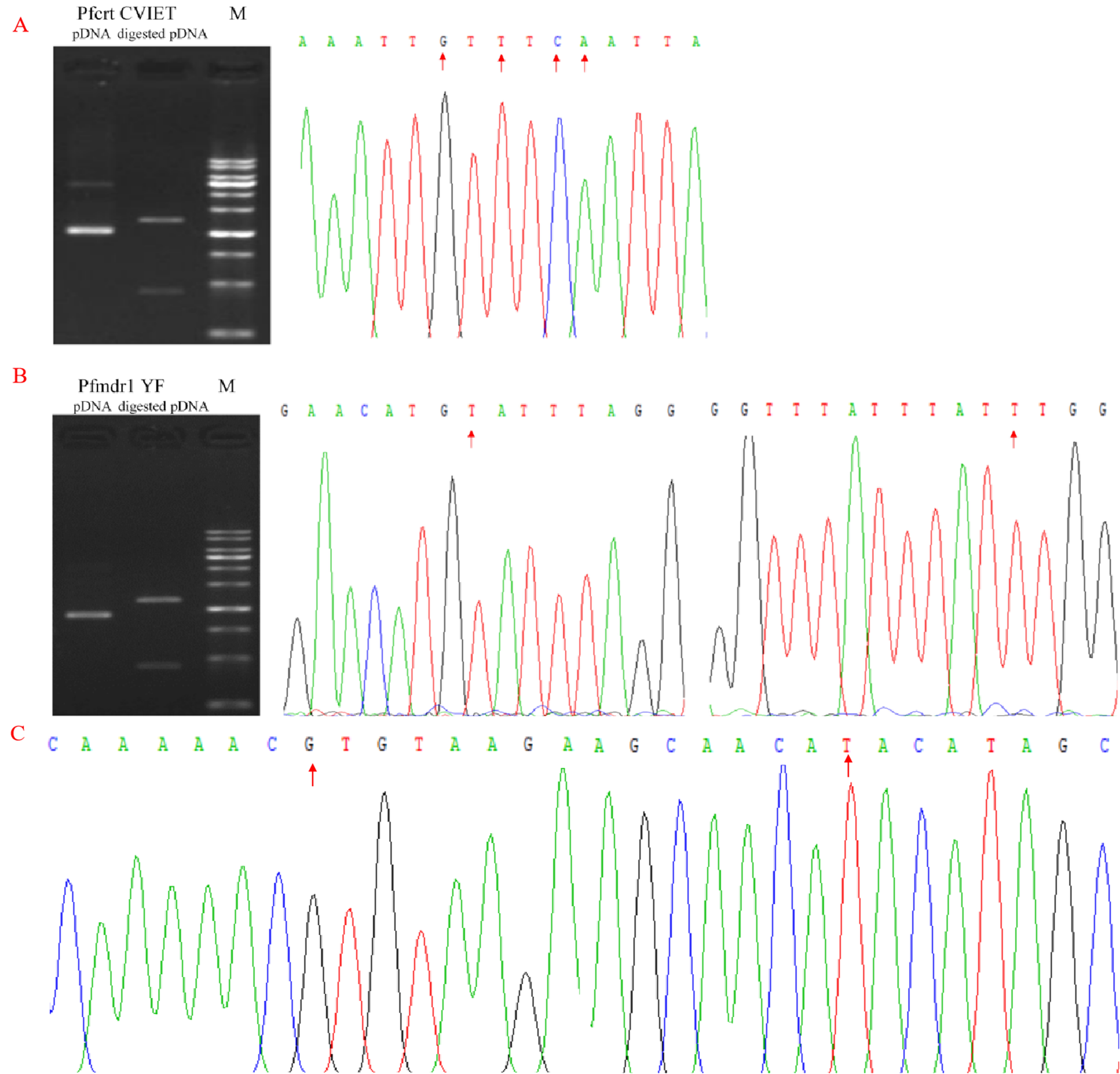

Supplement: Supplementary file 1 — Additional file 1: Fig. S1. Positive controls. A. pfcrt CVIET plasmid running in agarose gel electrophoresis and product sequence analysis; B. pfmdr1 NY plasmid with mutations at 86 and 184 sites running in agarose gel electrophoresis and product sequence analysis. C. Sequencing profile of pfk13 plasmid (JPG 3459 KB) [file 12936_2021_3737_MOESM1_ESM.jpg]
